# Quasi-two-dimensional α-molybdenum oxide thin film prepared by magnetron sputtering for neuromorphic computing†

**DOI:** 10.1039/d2ra02652j

**Published:** 2022-06-15

**Authors:** Zhenfa Wu, Peng Shi, Ruofei Xing, Yuzhi Xing, Yufeng Ge, Lin Wei, Dong Wang, Le Zhao, Shishen Yan, Yanxue Chen

**Affiliations:** School of Physics, and State Key Laboratory of Crystal Materials, Shandong University Jinan 250100 China cyx@sdu.edu.cn; School of Microelectronics, and State Key Laboratory of Crystal Materials, Shandong University Jinan 250100 China wl2003@sdu.edu.cn; School of Electronic and Information Engineering, Qilu University of Technology Jinan 250353 China

## Abstract

Two-dimensional (2D) layered materials have attracted intensive attention in recent years due to their rich physical properties, and shown great promise due to their low power consumption and high integration density in integrated electronics. However, mostly limited to mechanical exfoliation, large scale preparation of the 2D materials for application is still challenging. Herein, quasi-2D α-molybdenum oxide (α-MoO_3_) thin film with an area larger than 100 cm^2^ was fabricated by magnetron sputtering, which is compatible with modern semiconductor industry. An all-solid-state synaptic transistor based on this α-MoO_3_ thin film is designed and fabricated. Interestingly, by proton intercalation/deintercalation, the α-MoO_3_ channel shows a reversible conductance modulation of about four orders. Several indispensable synaptic behaviors, such as potentiation/depression and short-term/long-term plasticity, are successfully demonstrated in this synaptic device. In addition, multilevel data storage has been achieved. Supervised pattern recognition with high recognition accuracy is demonstrated in a three-layer artificial neural network constructed on this α-MoO_3_ based synaptic transistor. This work can pave the way for large scale production of the α-MoO_3_ thin film for practical application in intelligent devices.

## Abstract 2

An all-solid-state synaptic transistor with about 4 orders conductance modulation is fabricated based on the α-MoO_3_ thin film. A three-layer artificial neural network with high recognition accuracy was constructed based on this synaptic transistor.

## Graphical abstract

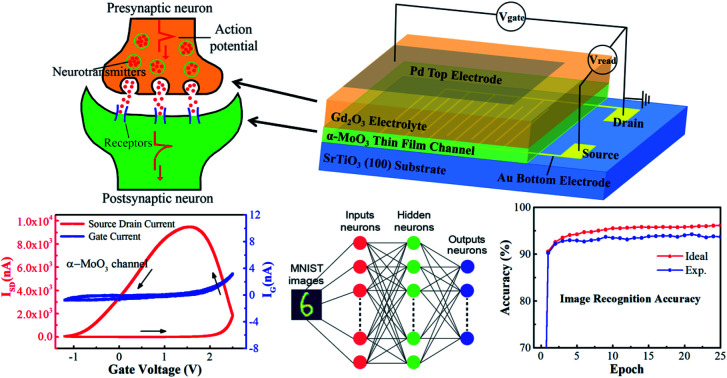

## Introduction

Today, the Internet of Things and artificial intelligence have brought great convenience to our lives in big data analytics, autonomous vehicles, speech and image recognition.^1,2^ However, they generate large quantities of complex data and present a huge challenge to the conventional von Neumann architectures. With the background of massive information, neuromorphic computing, seeking inspiration from the human brain to work in parallel and achieve high energy efficiency, hopes to solve computationally hard problems of the conventional computer systems.^3–5^ Synapses in the neuromorphic systems can combine information processing and memory by changing the synaptic weight.^1^ Interestingly, the electrolyte gated transistor (EGT) can modulate the conductance of the channel by ion migration, which mimics the function of biological synapses very well.^6^

For more than five decades, the traditional transistor has been shrinking exponentially in size in order to increase the switching speed, reduce the power dissipation and improve the integration density.^7^ However, scaling down of the transistor is approaching the limitation of miniaturization due to short-channel effects.^8^ An alternative solution to solve this bottleneck is using atomically thin semiconductor films, such as 2D materials, as the channel layer of the field effect transistor (FET).^9–15^ α-Molybdenum oxide (α-MoO_3_), has the well-known layered crystal structure, offers the possibility to obtain ultra-thin film.^16^ The layered α-MoO_3_ has unit cell parameters of *a* = 3.96 Å, *b* = 13.86 Å, *c* = 3.70 Å, which composed of double layers of linked and distorted MoO_6_ octahedra, and belongs to the space group *Pbnm*.^17,18^ α-MoO_3_ has been studied as the channel layer of synaptic transistor for neuromorphic computing in recent works.^19,20^ Unfortunately, in these works, the α-MoO_3_ film was prepared by the mechanical exfoliation, which could not realize large scale production for practical application. Wang *et al.* have fabricated α-MoO_3_ based two-terminal memristive device prepared by pulsed laser deposition (PLD).^21^ However, in their device, the thickness of α-MoO_3_ is 400 nm, which loses the excellent characteristics of 2D material based device. Moreover, the signal transmission and learning functions could not be carried out simultaneously in two-terminal memristive device.^19^ Therefore, large scale production of α-MoO_3_ thin film for practical application in neuromorphic computing needs further research.

In this work, an all-solid-state synaptic transistor based on α-MoO_3_ thin film prepared by magnetron sputtering is designed and studied. The surface morphology, crystal structure and valence characterizations indicate that a highly uniform orthorhombic single phase α-MoO_3_ thin film is obtained. About four orders of reversible conductance modulation is observed in the α-MoO_3_ channel by the protons (H^+^) intercalation/deintercalation under the positive/negative gate voltages. The essential synaptic functions, including potentiation, depression and short-term/long-term plasticity were successfully mimicked. High recognition accuracy is achieved using a simulated artificial neural network built from this α-MoO_3_ synaptic device. Overall, our study paves the way for large scale production of α-MoO_3_ thin film for practical application in intelligent devices.

## Experimental section

### α-MoO_3_ thin film preparation and characterizations

A Mo metal target (99.99%) was used to prepare α-MoO_3_ thin film on SrTiO_3_(100) single crystalline substrate (MTI, Hefei) at 450 °C by radio-frequency magnetron sputtering. The working pressure was 0.65 Pa (O_2_ 23% + Ar 77%) in a gas flow of 19.0 sccm. The sputtering power was 45 W and the deposition rate was about 0.1 Å s^−1^. The thickness of α-MoO_3_ thin film was about 18 nm. The surface morphology of α-MoO_3_ thin film was checked by atomic force microscope (AFM, Solver P47 PRO, NT-MDT) and scanning electron microscope (SEM, G300 FE-SEM System). The crystal structure and thickness of α-MoO_3_ thin film were characterized by X-ray diffraction (XRD, Smartlab, Rigaku Co). The thickness of α-MoO_3_ thin film was also characterized by a step profiler (Kosaka, ET 150). The chemical composition and bonding states of the α-MoO_3_ thin film were determined by X-ray photoelectron spectroscopy (XPS, Escalab 250).

### Device fabrication

After photolithography patterning, the bottom Cr/Au(3 nm/20 nm) source/drain electrodes were prepared on SrTiO_3_(100) substrate by thermal evaporation and lift-off method. The distance between source and drain electrodes was 20 μm. Then the α-MoO_3_ thin film used as the channel was deposited on the electrodes. And 30 nm amorphous Gd_2_O_3_ film was deposited on α-MoO_3_ thin film used as the electrolyte by radio-frequency magnetron sputtering at room temperature. During the deposition of Gd_2_O_3_ film, the working pressure was 0.65 Pa (O_2_ 50% + Ar 50%) in a gas flow of 19.0 sccm, and the sputtering power was 70 W. Finally, 6 nm Pd film was deposited on Gd_2_O_3_ film through a Cu hard mask as the top gate electrode by direct-current magnetron sputtering at room temperature. In this process, an all-solid-state synaptic transistor device is successfully fabricated.

### Electrical measurement

The transfer curves of the α-MoO_3_ based synaptic transistor device were measured using a custom-designed 4-probe station with two Keithley 2400 source meters under air and vacuum (<2 × 10^−4^ Pa) conditions, respectively. The voltage range was from −1.2 V to 2.5 V with a sweep rate of 2 mV s^−1^. The synaptic plasticity of the device was characterized by the 4-probe station in air condition.

## Results and discussion

### Structure and composition of α-MoO_3_ thin film

**Fig. 1:**
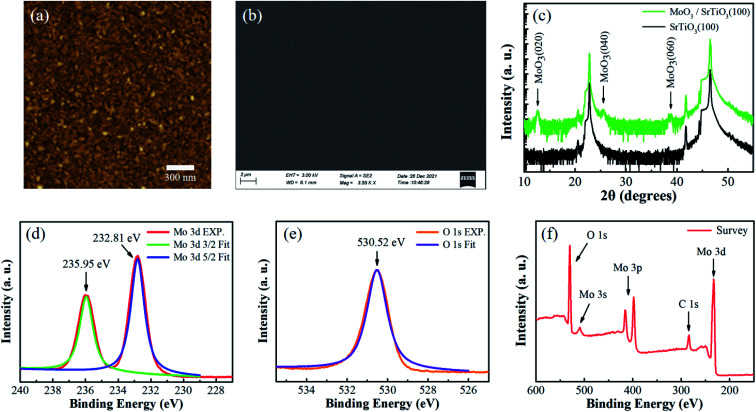
The properties of α-MoO_3_ thin film on SrTiO_3_(100) substrate. (a) AFM image of the α-MoO_3_ thin film, the scale bar is 300 nm. (b) SEM image of the α-MoO_3_ thin film, the scale bar is 2 μm. (c) XRD characterization of the α-MoO_3_ thin film and the SrTiO_3_ substrate. (d–f) XPS spectra of the α-MoO_3_ thin film. XPS spectra corresponding to the Mo 3d (d) and O 1s (e) core level of the α-MoO_3_ thin film. (f) The overview XPS result (150–600 eV) of the α-MoO_3_ thin film.

In this work, layered α-MoO_3_ thin film was prepared on SrTiO_3_ (100) single crystalline substrate by magnetron sputtering.^22^Fig. 1a exhibits a 2 × 2 μm AFM image (the scale bar is 300 nm) of the α-MoO_3_ thin film, which demonstrates a uniform surface. The root mean square (RMS) roughness is 1.31 nm, indicating that the film is smooth. As deduced from X-ray reflectance spectrum (Fig. S1a, ESI†), the thickness of the α-MoO_3_ thin film is about 18 nm (∼14 layers). The thickness of α-MoO_3_ thin film was also confirmed by a step profiler (Fig. S1c and d, ESI†). Fig. 1b shows the top-view SEM image (the scale bar is 2 μm) of this film, which demonstrates that the film is flat and continuous in the whole view. XRD characterization is carried out to study the microstructure of the α-MoO_3_ thin film. As shown in Fig. 1c, the peaks at 2*θ* = 12.7°, 25.6° and 38.9° correspond to the (020), (040) and (060) planes of α-MoO_3_ (JCPD: 05-0508), respectively.^18,19^ It is obvious that there is no impurity peak except the peaks of α-MoO_3_ thin film and SrTiO_3_(100) substrate in Fig. 1c. Because of its thin thickness, the intensity of α-MoO_3_ thin film peaks are relatively weaker than those of bulk α-MoO_3_ single crystal.^18^ The XRD results demonstrate that the α-MoO_3_ thin film is single orthorhombic phase with (010) preferred orientation.

The chemical composition and bonding states of the α-MoO_3_ thin film were characterized by XPS (Fig. 1d–f). The carbon C 1s peak at 284.6 eV was used to calibrate the XPS spectra. High-resolution scans of Mo 3d (Fig. 1d) and O 1s (Fig. 1e) were collected and fit with Gaussian–Lorentz distribution using a Shirley background. The binding energy peaks at 232.81 eV and 235.95 eV are the characteristic peaks of Mo 3d5/2 and Mo 3d3/2, respectively, which indicates that Mo in this film has a valence of +6. The binding energy peak at 530.52 eV is the characteristic peak of O 1s. All of these peaks are consistent well with previous reports.^23,24^ The total XPS spectrum (150–600 eV) of the α-MoO_3_ thin film is shown in Fig. 1f, in which the characteristic peaks of Mo 3d, C 1s, Mo 3p, Mo 3s and O 1s can be obtained. No peak related to impurity elements appears in the overview spectrum. In short, all the above results indicate high quality single phase α-MoO_3_ thin film was obtained on SrTiO_3_(100) substrate by magnetron sputtering.

### Electrical properties of α-MoO_3_ based synaptic transistor

**Fig. 2:**
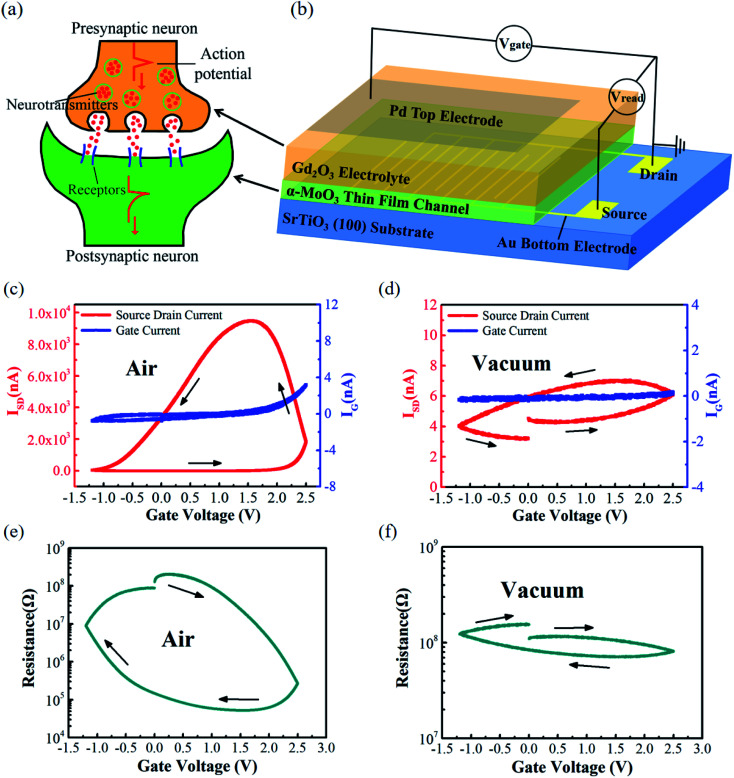
The schematic diagram of (a) the biological synapse and (b) the device structure. The transfer curves of α-MoO_3_ based synaptic transistor measured under (c) air and (d) vacuum conditions, respectively. The gate voltage window was from −1.2 V to 2.5 V, and the sweeping rate was 2 mV s^−1^. (e) and (f) show the channel resistance variations during sweeping the gate voltage under air and vacuum conditions, respectively.

Recently, electrolyte gated synaptic transistors have been studied extensively for neuromorphic computing due to their excellent performance.^6,14,25^ In this study, an all-solid-state synaptic transistor based on α-MoO_3_ thin film is successfully fabricated. Fig. 2a and b show the schematic diagrams of the biological synapse and the α-MoO_3_ based synaptic transistor. The biological synapse is composed of presynaptic neuron, neurotransmitters, synaptic cleft and postsynaptic neuron (Fig. 2a). After an action potential arrived at presynaptic neuron, the neurotransmitters will be released from presynaptic neuron into postsynaptic neuron through combination with specific receptors, resulting in variations of the postsynaptic potential to transmit information.^26–29^ Imitating the biological synapse, Gd_2_O_3_ solid-state electrolyte and α-MoO_3_ channel are used in the transistor device as the presynaptic neuron and postsynaptic neuron, respectively (Fig. 2b). In this artificial synaptic device, the external stimulus (voltage pulse) applied on the gate electrode will lead to hydrolysis reaction at the interface between Gd_2_O_3_ electrolyte layer and top Pd electrode, and create a lot of protons (H^+^) that function as neurotransmitters. These neurotransmitters (H^+^) will be driven across the Gd_2_O_3_ electrolyte and injected into α-MoO_3_ channel under a high enough positive gating voltage. As a result, the channel conductance (synaptic weight) will be modulated to transmit information.

In order to characterize the performance of the transistor, the transfer curves were measured in air (Fig. 2c) and under vacuum condition (Fig. 2d), respectively. The gate voltage (*V*_G_) was swept from 0 V to 2.5 V, 2.5 V to −1.2 V and then back to 0 V. The sweep rate was 2 mV s^−1^ and the source drain voltage (*V*_read_) was 0.5 V. The red and blue lines represent source drain current (*I*_SD_) and gate current (*I*_G_), respectively. As we can see, *I*_SD_ increases significantly (about 10^4^ times) and shows a clear anti-clockwise hysteresis by sweeping the *V*_G_ in air condition (Fig. 2c). When *V*_G_ was swept to the positive part, H^+^ ions will be injected into the MoO_3_ channel, resulting in H_*x*_MoO_3_ with a high conductance state. This process can be described by the following reaction:^16^

1 MoO_3_ + *x*H^+^ + *x*e^−^ → H_*x*_MoO_3_

In contrast, when *V*_G_ is reduced, H^+^ ions will be extracted from the H_*x*_MoO_3_ channel to Gd_2_O_3_ film, recovering the low conductance in MoO_3_ channel. This process can be described by the following reaction:

2 H_*x*_MoO_3_ → MoO_3_ + *x*H^+^ + *x*e^−^

The magnitude of *I*_G_ is below 4 nA during the whole sweeping cycle, which is more than three orders smaller than *I*_SD_, indicating that the leakage current has little effect on *I*_SD_. However, if the air was pumped out of the probe station chamber and the transfer curve was obtained under vacuum condition, *I*_SD_ increases slightly and shows a tiny anti-clockwise hysteresis by sweeping the *V*_G_ (Fig. 2d). The red and blue lines represent *I*_SD_ and *I*_G_, respectively. The dramatically different gating responses observed in air and vacuum conditions indicate that the water molecular in air may play a crucial role in modulating the channel conductance.^19,30,31^ In addition, some theoretical calculations performed with the density functional theory have also predicted that the conductance of MoO_3_ can be changed by hydrogenation.^32–34^ The resistance data of the α-MoO_3_ channel were extracted from the *I*_SD_–*V*_G_ curves, as shown in Fig. 2e and f. The channel resistance changes from 10^8^ Ω to 10^4^ Ω and exhibits a clear clockwise hysteresis by sweeping the *V*_G_ in air condition, while it changed slightly under vacuum condition. The slight channel resistance variation under vacuum condition is caused by residual H^+^ ions in the device.

### Synaptic plasticity of the synaptic transistor

**Fig. 3:**
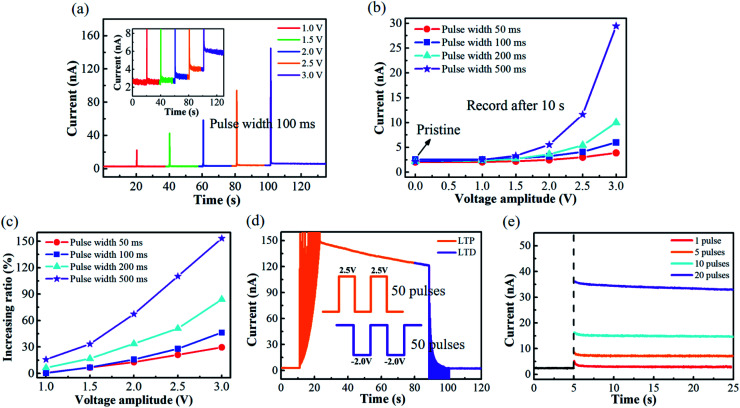
(a) The channel conductance variations under different *V*_G_ (1.0 V–3.0 V) with a fixed pulse width (100 ms). The inset shows the detailed conductance variations after each spike. (b) The channel conductance values (record after 10 s of each spike) and (c) the increasing ratio of channel conductance under different *V*_G_ (1.0 V–3.0 V) with different pulse width (50 ms–500 ms). (d) Long-term potentiation (2.5 V, 100 ms, 50 pulses) and long-term depression (−2.0 V, 100 ms, 50 pulses). (e) Spike number dependent plasticity (SNDP). The pulse numbers increase from 1 to 20 with a fixed *V*_G_ (2.0 V, 100 ms).

The obvious hysteresis in the transfer curve of this novel transistor lays a good foundation for potential synaptic application. To demonstrate the nonvolatile characteristics, the solid-state α-MoO_3_ transistor was trained by sending a series of voltage pulses to the gate electrode. During the voltage pulses, the source-drain current is recorded at the same time. The synaptic plasticity characteristics of the artificial synaptic transistor device are shown in Fig. 3a. A series of single voltage pulse stimulation with different magnitudes (1.0 V–3.0 V) and identical pulse width of 100 ms were applied to gate electrode, and a source-drain voltage of 0.5 V was applied. Similar to the short-term plasticity (STP) in biological synapse, *I*_SD_ shows a sharp peak response to the *V*_G_, and quickly decreases to the initial value under low *V*_G_ of 1.0 V and 1.5 V. While under a higher *V*_G_ (>2.0 V), the *I*_SD_ could not return to the initial value after the gate stimulation, indicating a nonvolatile behavior. This means that a memory behavior can be realized using higher voltage gating pulses. A more detailed study of the synaptic plasticity characteristics under different *V*_G_ (pulse magnitude: 1.0–3.0 V, pulse width: 50–500 ms) was shown in Fig. 3b and c. These values of *I*_SD_ were recorded after 10 s of each spike. It can be clearly seen that the channel conductance change increases with the pulse width and amplitude of *V*_G_. Voltage pulses with longer duration time and larger magnitude can lead to a higher H^+^ doping in the α-MoO_3_ channel, resulting in a larger nonvolatile change of the channel conductance. The energy consumption in plasticity process was estimated. The energy consumption per spike is about 5.0 × 10^−10^ J as calculated with the formula *I*_peak_ × Δ*t* × *V*_read_,^35^ where *I*_peak_, Δ*t* and *V*_read_ represent the peak value of the *I*_SD_ (∼20 nA), the pulse width (50 ms) and the source drain voltage (0.5 V), respectively. The energy consumption can be further reduced by device miniaturization for practical application.

It is well known that bidirectional plasticity, that is the long-term potentiation (LTP) and long-term depression (LTD), is a key concept in synapses. Potentiation and depression represent synaptic strengthening and weakening, respectively. These functions were mimicked in our synaptic transistor device. The channel conductance (synaptic weight) can be reversible modulation by insertion/extraction of H^+^ ions into/from the α-MoO_3_ thin film under the positive/negative electric bias. Fig. 3d shows the LTP and LTD of the synaptic transistor. By alternatively applying 50 identical pulses (2.5 V, 100 ms), *I*_SD_ gradually increases with the positive gate spikes (LTP), while *I*_SD_ decreases to the initial value (LTD) after 50 negative gate spikes (−2.0 V, 100 ms). Fig. 3e depicts the spike number dependent plasticity (SNDP) of the synaptic transistor, which was measured by monitoring the channel conductance after applying 1, 5, 10 and 20 identical pulses (2.0 V, 100 ms). It is obvious that the *I*_SD_ increases with the pulse numbers. Fig. 3e also shows that after the withdrawal of the gate spikes, the channel current decays slowly with time, just like the forgetting process in biological systems. Fig. 3d and e also demonstrate a nonvolatile behavior of the synaptic transistor. However, if the decaying time is much longer than the switching time, this decaying process will have little effect on the operation of this synaptic device.

### Analog switching of the synaptic transistor

**Fig. 4:**
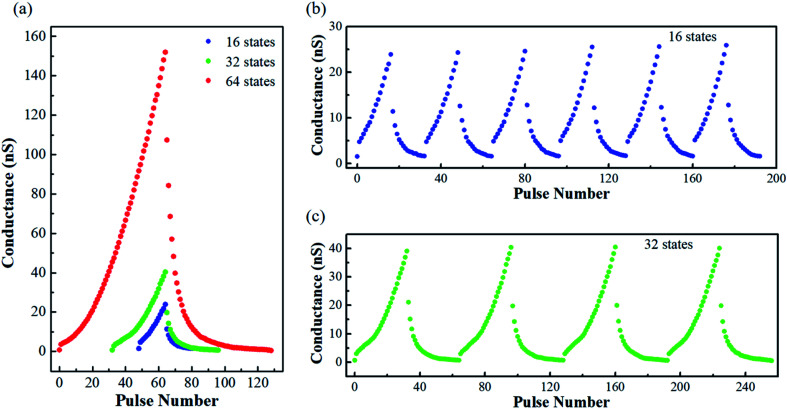
Analog switching of the synaptic transistor. (a) 16/32/64 multi-level states obtained by applying 16/32/64 positive and negative *V*_G_ pulses. The values of channel conductance were read under a *V*_read_ of 0.5 V. (b) and (c) Cycle testing with 16 and 32 positive/negative *V*_G_ pulses, respectively.

As shown above, several essential synaptic functions have been realized in the α-MoO_3_ based synaptic transistor device. Realization of multi-states in synaptic weight is a requisite condition for artificial neuromorphic computing. Fig. 4a shows the 16/32/64 multi-level data storage functions obtained by applying 16/32/64 positive (2.0 V, 100 ms) and negative (−2.0 V, 100 ms) *V*_G_ pulses. The values of channel conductance were calculated with *I*_SD_ divided by *V*_read_. The *G*_max_/*G*_min_ ratios of the 16/32/64 multiple states are as large as 15.9/55.2/183.0, respectively, where the *G*_max_ and *G*_min_ represent the maximum and the minimum (initial) channel conductance values, respectively. Large *G*_max_/*G*_min_ ratio can provide a potential opportunity to obtain more storage states.

To demonstrate the behaviors of weight update, a repeated 16/32 potentiation and depression cycling test was performed, as shown in Fig. 4b and c. The transistor device exhibits good repeatability and stability. To check the linearity and stability of the weight update behavior during analog switching, the asymmetric ratio (AR) and cycle-to-cycle variation (C2C) were calculated, respectively. The AR can be obtained using the following formula:^20,37,38^3
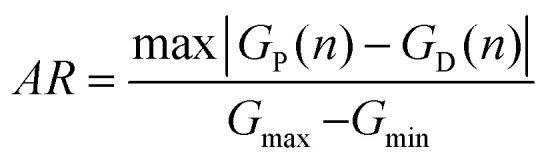
Where *n* (from 1 to 32) is the voltage pulse number of the 32 potentiation and depression cycle, *G*_P_(*n*) and *G*_D_(*n*) are the channel conductance values at *n*th state during the potentiation and depression processes, respectively. *G*_max_ and *G*_min_ are the maximum and minimum channel conductance values, respectively. The value of AR is zero for ideal linear device. Here, the calculated AR of the α-MoO_3_ based synaptic transistor device is 0.58 ± 0.0069, which is comparable to previous report.^37^ The linearity of our device still needs to be further improved for achieving higher image recognition accuracy, which will be discussed in the next section. The cycle-to-cycle variation (the average of the channel conductance standard deviation divided by the maximum conductance)^20,26,27^ was characterized with 5 sequential switching cycles. It can be defined as:4
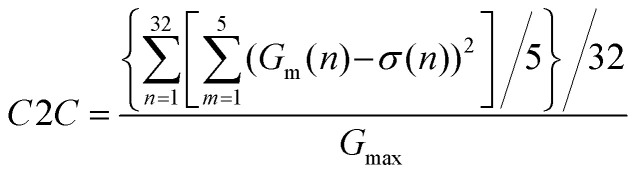
Where *m* (from 1 to 5) is the number of cycles, *n* (from 1 to 32) is the voltage pulse number of each cycle. σ(*n*) is the mean value of channel conductance in *n*th state, which is the average of channel conductance of *n*th voltage pulse in 5 switching cycles. *G*_m_(*n*) is the channel conductance of *m*th cycle in *n*th state. *G*_max_ is the maximum value of channel conductance. The calculated C2C of the α-MoO_3_ based synaptic transistor device is as low as 1.02%. This low C2C demonstrates a low write noise during neuromorphic computing.

### Simulation of supervised pattern recognition

**Fig. 5:**
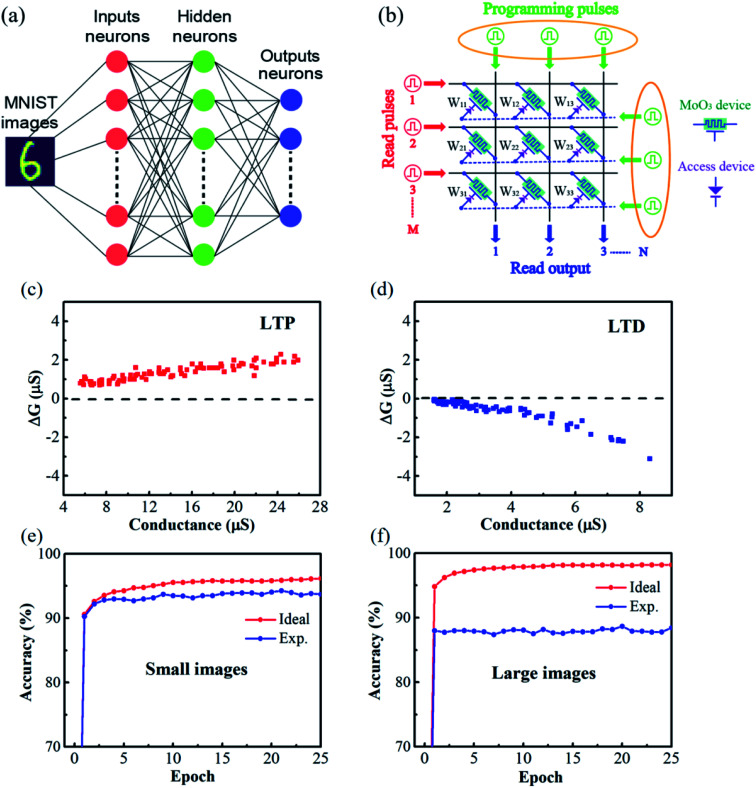
The simulation of supervised learning in the α-MoO_3_ based synaptic transistor device. (a) Schematic image of a three-layer (one hidden layer) artificial neural network. (b) Schematic image of the crossbar array made up of α-MoO_3_ based transistor. (c and d) The changes in channel conductance (Δ*G*) in (c) LTP and (d) LTD processes. (e and f) The recognition accuracy for (e) small images and (f) large images with an ideal device and α-MoO_3_ based device.

Finally, the computing performance of the α-MoO_3_ based synaptic transistor was evaluated. As shown in Fig. 5a, a three-layer artificial neural network (one hidden layer) was constructed to perform supervised learning based on a back-propagation algorithm. The neurons of every layer connect with each other. Back-propagation is a widely used method for training artificial neural networks in neuromorphic computing.^39^ Two data sets were employed to train this artificial neural network: a small image version (8 × 8 pixels) of handwritten digits from the “Optical Recognition of Handwritten Digits” data set,^40^ and a large image version (28 × 28 pixels) of handwritten digits from the “Modified National Institute of Standards and Technology” (MNIST) data set.^41^Fig. 5b shows the crossbar array of the CrossSim simulator, which was used to perform vector-matrix multiplication and outer-product update operations.^36,42^ The crossbar array contains read pulses (red color), programming pulses (green color) and read outputs (blue color). The α-MoO_3_ channel conductance represents the synaptic weight.

The LTP/LTD experimental data in Fig. 4b were selected for supervised simulation. The simulating results were demonstrated in Fig. 5c–f. The channel conductance deviation (Δ*G*) between experimental values were characterized to evaluate the device nonideality. In contrast to C2C, Δ*G* is the change of channel conductance induced by single voltage pulse within each cycle. As we can see, the distribution of Δ*G* in LTP process (Fig. 5c) is narrower than that in LTD process (Fig. 5d) over the entire range of *G*. The image recognition accuracy of the simulated network after training 25 epochs was plotted in Fig. 5e and f. High image recognition accuracies of 94% for small images (Fig. 5e) and 88% for large images (Fig. 5f) were obtained. Although the recognition accuracy is comparable to recently reported results in α-MoO_3_ based synaptic devices,^19–21^ further works are still required to optimize the linearity and symmetry of analogy switching in this device to increase the image recognition accuracy. Finally, the properties of our α-MoO_3_ synaptic transistor were compared with those of other devices reported in the literature, which were summarized in Table 1.^19–21,26,27,43–45^ Our solid-state device shows a high stability and good recognition accuracy. What's more, no ionic liquid and organic material is adopted in our device, which make it more compatible with current semiconductor processes.

**Table 1:** Summary of the fabrication methods and synaptic characteristics of synaptic transistor devices

Device structure	Active ions	Channel fabrication method	Channel thickness [nm]	Stability	Energy consumption [J]	Recognition accuracy	Ref.
Gd_2_O_3_/α-MoO_3_/SrTiO_3_	H^+^	Sputtering	18	1.02%	<5.0 × 10^−10^	94%	This work
IL^a^/α-MoO_3_/SiO_2_/Si	H^+^	Mechanical exfoliation	12.6	—	9.6 × 10^−12^	—	19
SL^b^/α-MoO_3_/SiO_2_/Si	Li^+^	Mechanical exfoliation	18	6.5%	1.6 × 10^−13^	94%	20
Pt/α-MoO_3_/Nb–SrTiO_3_ (two terminal device)	H^+^	PLD^c^	400	1.4%	—	—	21
IL^a^/VO_2_/Mica	H^+^	PLD^c^	40	2.1%	8.8 × 10^−13^	95%	26
SL^b^/VO_2_/Polyimide	H^+^	Sputtering	51	0.17%	<6.2 × 10^−9^	98%	27
MoS_2_/Na–SiO_2_/Si	Na^+^	Mechanical exfoliation	10	—	∼3.0 × 10^−7^	90%	43
MoTe_2_/sr-SiN_*x*_/Si	e^−^/holes	Mechanical exfoliation	7	1.5%	∼1.0 × 10^−10^	91%	44
α-In_2_Se_3_/Ta_2_O_5_/P–Si	e^−^/holes	Mechanical exfoliation	30	—	1.0 × 10^−11^	93%	45

a Ionic liquid.

b Solid state ionic liquid.

c Pulsed laser deposition.

## Conclusion

In summary, an all-solid-state synaptic transistor based on single phase α-MoO_3_ thin film prepared by magnetron sputtering is designed and fabricated. Four orders of reversible conductance modulation is realized experimentally in the α-MoO_3_ thin film by H^+^ ions intercalation/deintercalation. Several essential synaptic behaviors in biological synapse, including STP, LTP/LTD and SNDP are successfully mimicked in our device. An artificial neural network based on α-MoO_3_ based synaptic transistor was constructed to perform supervised learning. High recognition accuracies of 94% and 88% were achieved for Handwritten Digits data set (small images) and MNIST data set (large images), respectively. In addition, both channel material and electrolyte material in this device are prepared by magnetron sputtering, which is compatible with modern semiconductor technology. This work can pave the way for large scale production of quasi-2D material for practical application in synaptic transistor device.

## Conflicts of interest

There are no conflicts to declare.

## Supplementary Material

RA-012-D2RA02652J-s001
