# Supplementary material for: Quasi-two-dimensional α-molybdenum oxide thin film prepared by magnetron sputtering for neuromorphic computing
Source: RSC Adv. 2022 Jun 15;12(28):17706–14. doi: 10.1039/d2ra02652j (PMC9199084; doi:10.1039/d2ra02652j)
Supplement: RA-012-D2RA02652J-s001 [file RA-012-D2RA02652J-s001.pdf]

Electronic Supplementary Material (ESI) for RSC Advances.
This journal is © The Royal Society of Chemistry 2022

Supporting Information

Quasi-Two-Dimensional α-Molybdenum Oxide Thin Film Prepared by Magnetron Sputtering for Neuromorphic Computing

Zhenfa Wu,[a] Peng Shi,[a] Ruofei Xing,[a] Yuzhi Xing,[a] Yufeng Ge,[a] Lin Wei,*[b] Dong Wang,[a] Le Zhao,[c] Shishen Yan[a] and Yanxue Chen*[a]

[a]*School of Physics, and State Key Laboratory of Crystal Materials, Shandong University, Jinan 250100, China*

[b]*School of Microelectronics, Shandong University, Jinan 250100, China*

[c]*School of Electronic and Information Engineering, Qilu University of Technology, Jinan 250353, China*

* *Corresponding author. E-mail: cyx@sdu.edu.cn (Y. X. Chen), wl2003@sdu.edu.cn (L. Wei)*

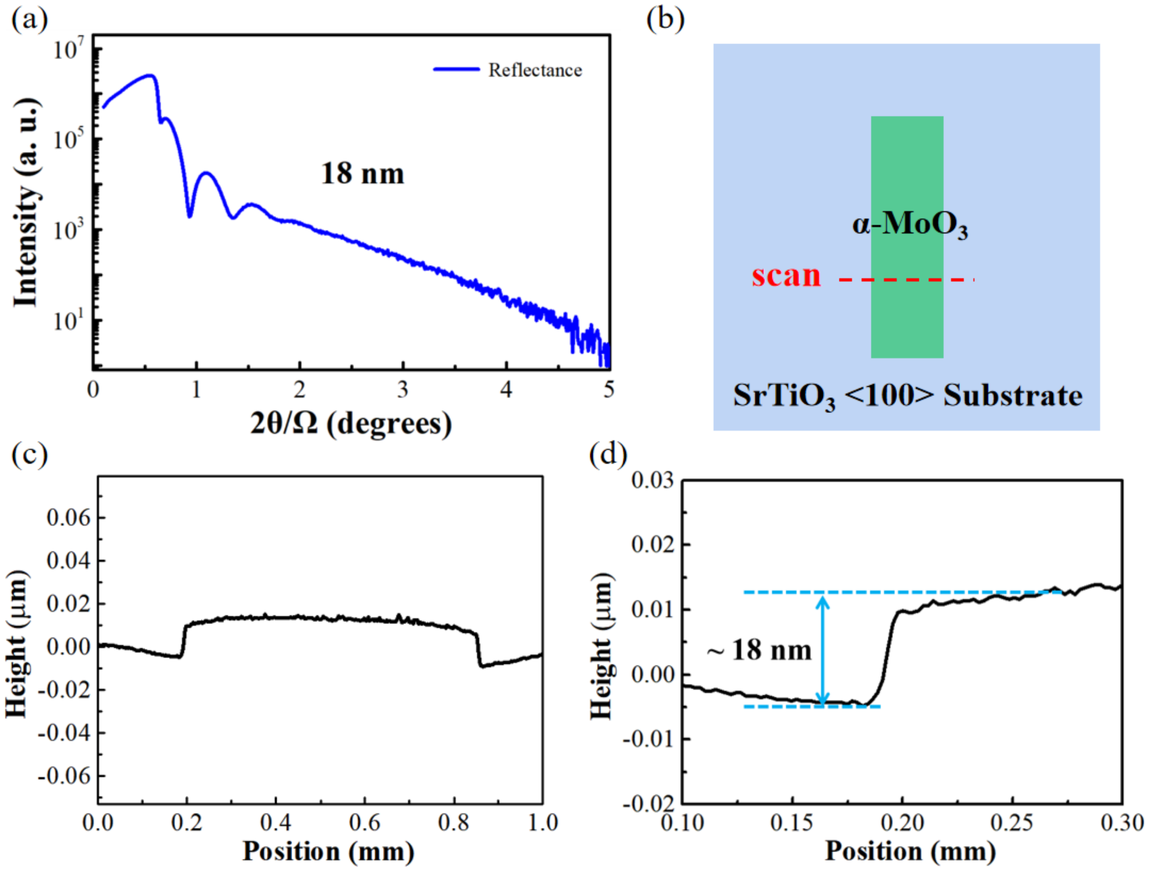

Fig. S1. The thickness characterization of α-MoO_3 thin film. (a) The X-ray reflectance spectrum of α-MoO_3 thin film. (b) Schematic image of the α-MoO_3 bar grown on $SrTiO_3$ substrate through a hard mask. (c, d) The step profiler spectrum of the α-MoO_3 bar.
